# Simulation and experimental evaluation of laser-induced graphene on the cellulose and lignin substrates

**DOI:** 10.1038/s41598-024-54982-1

**Published:** 2024-02-23

**Authors:** Ali Ghavipanjeh, Sadegh Sadeghzadeh

**Affiliations:** School of Advanced Technologies, Iran University of Science and Technology, Tehran, Iran

**Keywords:** Laser-induced graphene, MD simulation, Cellulose, Lignin, Engineering, Materials science, Physics

## Abstract

In this article, the formation of laser-induced graphene on the two natural polymers, cellulose, and lignin, as precursors was investigated with molecular dynamics simulations and some experiments. These eco-friendly polymers provide significant industrial advantages due to their low cost, biodegradability, and recyclable aspects. It was discovered during the simulation that LIG has numerous defects and a porous structure. Carbon monoxide, H_2_, and water vapor are gases released by cellulose and lignin substrates. H_2_O and CO are released when the polymer transforms into an amorphous structure. Later on, as the amorphous structure changes into an ordered graphitic structure, H_2_ is released continuously. Since cellulose monomer has a higher mass proportion of oxygen (49%) than lignin monomer (29%), it emits more CO. The LIG structure contains many 5- and 7-carbon rings, which cause the structure to have bends and undulations that go out of the plane. In addition, to verify the molecular dynamics simulation results with experimental tests, we used a carbon dioxide laser to transform filter paper, as a cellulose material, and coconut shell, as a lignin material, into graphene. Surprisingly, empirical experiments confirmed the simulation results.

## Introduction

Graphene is a two-dimensional sheet of sp2 carbon atoms arranged in a hexagon structure^1^. Due to its excellent mechanical^2,3^, electrical^4,5^, and thermal properties^6,7^, graphene has many advantages in engineering. Graphene is manufactured by various methods, such as chemical vapor deposition (CVD)^8^ and mechanical exfoliation^1^, and its features and applications vary depending on the manufacturing method. Most graphene production techniques require high temperatures, long synthesis times, or high costs^9,10^.

Among the promising methods for graphene production, laser-induced graphene (LIG)^11^ has received significant attention due to its potential for cost-effective, large-scale graphene synthesis^11^. It is a simple, rapid, and chemical-free process for producing graphene^12^ and has led to graphene with a highly porous structure and superior resistance^13^.

LIG is produced by irradiating a carbon material substrate with a laser, which induces the formation of graphene flakes on the substrate's surface. Tour and his colleagues used a CO2 laser (10.6 µm) to perform this process on a polyamide layer^13^. Later, the same group and others achieved this operation on other synthetic materials like PEEK, PC, and PEI^13^, as well as on natural materials including, silk^14^, lignin^15,16^, wood^17,18^, Xylan^19^, paper^18–20^, coconut shell and potato skin^18^. LIG has several uses in various Sensors^14,19–25^, micro-supercapacitors^10,16,26^, and Batteries^27,28^.

The mechanism of LIG formation is believed to involve the carbonization of the substrate material under laser irradiation, Photons quickly heat the precursor and gather energy from it, providing a high-pressure, high-temperature reaction process^13,29^. At temperatures around 2500 degrees Celsius, sp3 carbons are converted to sp2 carbons^13^, functional groups are removed, and bonds between atoms such as C–C and C–H or those in aromatic compounds are broken^30^.

One of the main types of used natural precursors is Lignocellulosic materials which are composed of three base polymers: cellulose, hemicelluloses, and lignin^31^. Studies and the use of these precursors in the synthesis of LIG have found many advantages due to their low cost, biodegradability, and recyclable aspects. Compared to synthetic materials, natural materials provide significant industrial advantages^19,32,33^.

Both cellulose and lignin are the main components of plant cell walls^34^, and they constitute a significant portion of the Earth's biomass^31,35^. Lignin is a complex, amorphous polymer that provides structural support to plant cell walls and is often considered a waste material in the pulp and paper industry^34,36^. Cellulose, on the other hand, is a linear polymer composed of glucose monomers that form long, crystalline fibrils and is one of the most widely used biomaterials in various applications ^37,38^.

In the first studies about LIG synthesis, Wood as a Lignocellulosic material transformed into LIG, under an Ar/H_2_ atmosphere because direct laser irradiation in air induced ablation of wood instead of LIG formation^17^. It has been observed that lignin content/structure, is one of the essential parameters for lignocellulosic LIG synthesis. Since lignin is rich in aromatic subunits that are easily transformed into graphene by heat treatment, woods with a high lignin content (such as pinewood) are more favorable for the production of high-quality LIG than woods with a low lignin concentration^17,18^.

In addition to Wood with lignin contents (typically 18–30% lignin), several natural materials having lignin components, including cork, coconut shells, and potato skins (≈ 25%, 30%, and 36% lignin, respectively)^39–41^. Under ambient conditions, repeated exposure to a low-power CO_2_ laser directly transformed These materials to LIG; in contrast, wood was burned up or volatilized under similar situations.

A multiple-lasing method was proposed to make the LIG process more widely applicable to a variety of substrates. Low-fluence pulses are used to transform the substrate to amorphous carbon, which is subsequently converted to LIG using a laser beam. Defocusing the laser beam was used to reduce laser fluence and increase the overlap between laser spots on the sample and it may be used to convert various polymers into Lig, this approach helps reduce laser ablation and thermal damage^18^.

Producing high-quality laser-induced graphene (LIG) on cellulose and lignin substrates requires the optimization of laser parameters. The laser power, scanning speed, and number of passes are crucial factors that affect the quality and morphology of LIG. If the laser power and scanning speed are too low, the formation of graphene will be incomplete. On the other hand, high power and fast speed can damage the substrate. The quality of LIG also depends on the number of passes, with a higher number resulting in thicker and more uniform graphene films. The substrate's surface roughness and chemical composition should also be taken into account since they can influence the laser energy's absorption and thermal conductivity^8,12,18,19^.

However, the exact mechanism of the LIG formation process is still a subject of debate. Some studies suggest that the formation of LIG is primarily due to laser-induced pyrolysis, while others propose that it is due to the formation of carbon radicals, which then polymerize to form graphene^11,42–45^. Molecular dynamic simulations in carbonization reactions and graphite structure formation afford many insights into the chemistry of these processes and their mechanisms.

LAMMPS^46^ (Large-scale Atomic/Molecular Massively Parallel Simulator) is a widely used open-source molecular dynamics simulation software that is designed to run efficiently on parallel computing architectures. It is a highly versatile software that can simulate a wide range of molecular systems, including biological molecules, polymers, and solid-state materials. LAMMPS uses a variety of interatomic potentials to model the interactions between atoms in a system, including classical force fields, quantum mechanical potentials, and hybrid potentials.

Conventional force fields, which rely on fixed functional forms to describe the interactions between atoms, have limitations when it comes to simulating chemical reactions. Reactive force fields such as ReaxFF have been developed to address this challenge^44,47^.

ReaxFF^47^ was developed in the 1990s by Adri van Duin, William Goddard, and co-workers. It is a force field that includes both reactive and non-reactive interactions between atoms. The reactive part of the force field allows for the simulation of bond breaking and formation, as well as other chemical reactions. The non-reactive part of the force field is used to describe the van der Waals and electrostatic interactions between atoms. ReaxFF has been used in the carbonization of soot, phenolic resins, cellulose, and polyamide.

**Figure 1:**
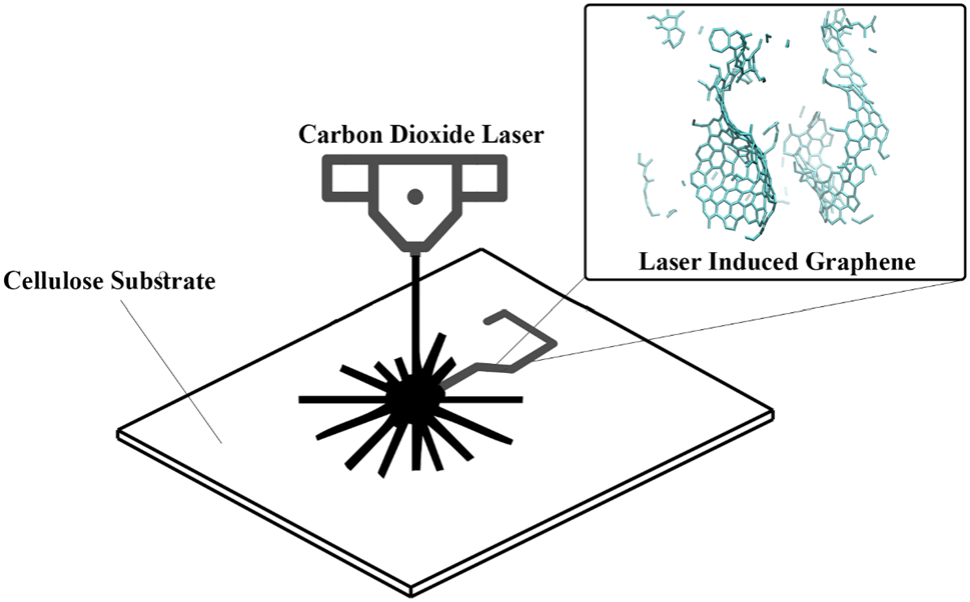
Schematic of the synthesis process of LIG on a cellulose substrate and an overview of the produced LIG from cellulose molecules with MD.

Vashtish et al.^42^ published excellent results in 2020 after studying the simulation of LIG formation on a variety of synthetic polymers with ReaxFF. In this study, we performed a molecular dynamics simulation with LAMMPS and REAXFF of the LIG production process based on two eco-friendly natural polymers: cellulose and lignin. Figure 1 illustrates the synthesis process of LIG on a cellulose substrate and the final produced LIG from cellulose molecules with MD.

## Modeling and simulation approach

### Model building

Cellulose (C_6_H_10_O_5_)n is a polysaccharide made up of hundreds to several thousands of 1,4-b-d-glucopyranose units joined by 1,4-d glycoside linkages. Cellulose macromolecules include a significant number of hydroxyl groups, which contribute to intramolecular and intermolecular hydrogen bonding^48^.

Lignin is an amorphous, highly branched, complex biopolymer composed of phenylpropane units linked by ether or C–C bond.

s. Both phenolic and aliphatic hydroxyl groups are present in lignin.

Lignin's precise chemical structure is difficult to characterize. Instead, a source-dependent linkage distribution is frequently reported^49,50^.

In this work, two separate unit cells of lignin (organosolv) and cellulose (1,4-d beta-glucose) molecules (each having ~ 1400 atoms) were made, and the system was taken under NVT(Nose Hoover Thermostat). The time step was set to 0.25 fs, and the initial system was prepared by following the steps.

**Figure 2:**
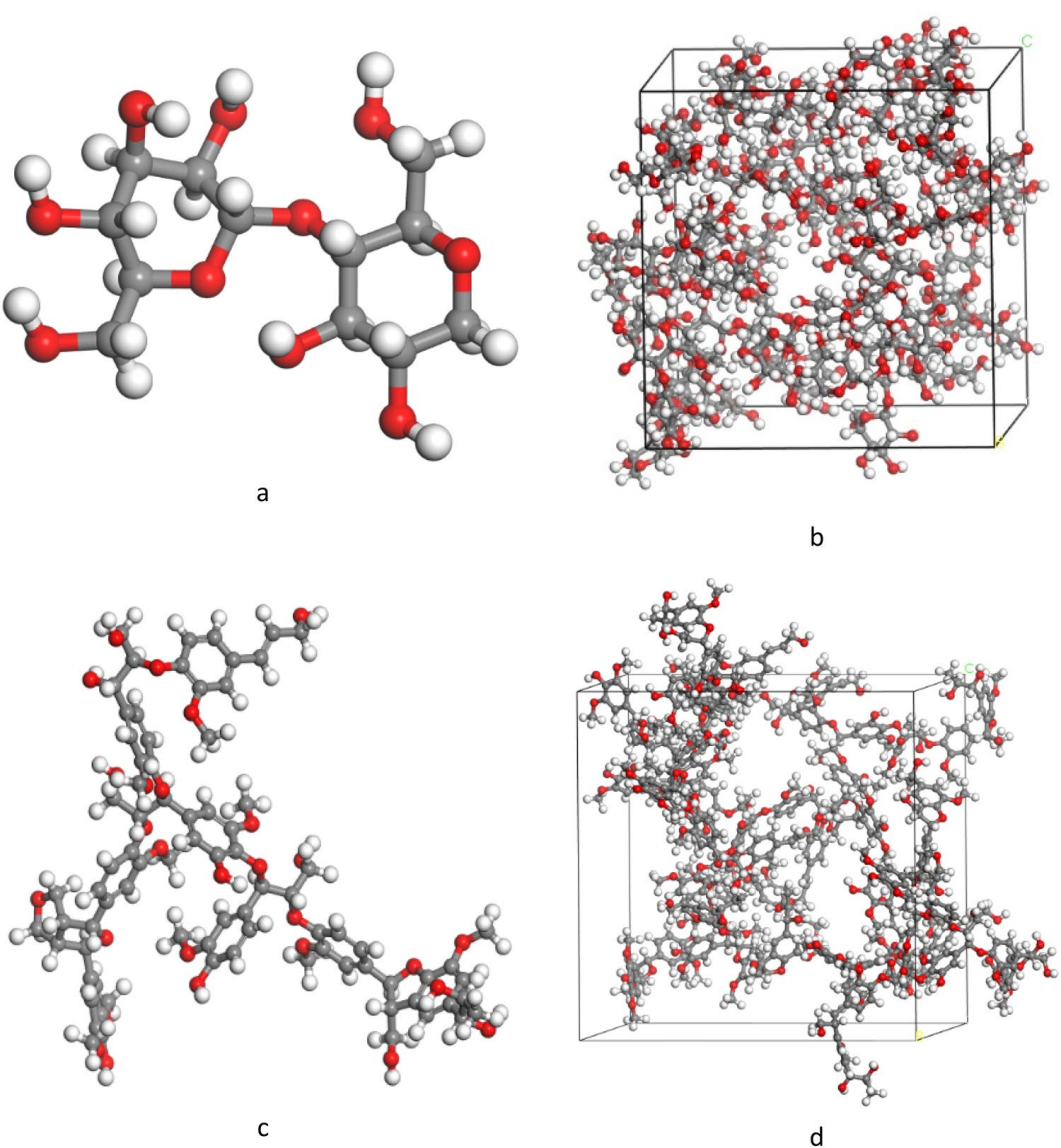
(**a**) 1,4-D B Glucose monomer. (**b**) cellulose simulation box. (**c**) lignin monomer. (**d**) lignin simulation box.

The design of the models begins with the construction and optimization of cellulose (1,4 b D glucose) and lignin monomers using the Materials Studio^51^ Forcite module and the universal force field. The molecular structure of the monomers that were used in the simulations is shown in Fig. 2a and c. Using the construction function of the Amorphous Cell module, this monomer was subsequently constructed into a cubic simulation cell and reached the desired density (1.07 g/cm^3^ for cellulose^52^ and 0.49 g/cm^3^ for lignin^50^). representative simulation cells are shown in Fig. 2b and d. After that, the system relaxed with NVT at 300 k, heated by NVT to 3000 K with a heat rate of 0.01 k/fs, and kept the system at this temperature for 2 ns. Figure S2 shows the molecular dynamic simulation stages. Also, videos showing the LIG structure are provided as movie S1 and movie S2 in supplementary information.

### ReaxFF method

ReaxFF^44,47^ is a reactive force field that has gained popularity due to its ability to simulate chemical reactions with high accuracy and computational efficiency. It forms or breaks bonds at every molecular dynamics iteration and all connectivity-related interactions are bond order dependent. The bond order is updated during the simulation as atoms move and interact with each other, determining the energy associated with the bond formation and bond breaking.

In addition to the bond-order formalism, ReaxFF includes a set of empirical parameters describing the interactions between different atom types. These parameters are determined by fitting the force field to experimental data or quantum mechanical calculations. The fitted parameters are specific to each chemical system and allow for accurate simulation of chemical reactions in that system.

Compared to quantum mechanical calculations, which can be very computationally expensive for large systems, ReaxFF simulations can be performed on relatively modest computing resources. ReaxFF is also able to capture the dynamics of chemical reactions, which is difficult to achieve with static quantum mechanical calculations.

This empirical potential divides the total inter-atomic energy into various partial energy contributions.

1 $${E}_{total}+={E}_{bond}+{E}_{under}{+ E}_{over}+{E}_{IonP}+{E}_{val}+{E}_{tor}+{E}_{vdWaals}+{E}_{Coloumb}+{E}_{Rest}$$

where ${E}_{bond}$ = bond energy, ${E}_{over}$ = over-coordination energy penalty, ${E}_{under}$ = under-coordination stability, ${E}_{IonP}$= lone pair energy, ${E}_{val}$= valence angle energy, ${E}_{tor}$= torsion angle energy, ${E}_{vdWaals}$ = van der Waals energy, ${E}_{Coloumb}$ =coulomb energy, ${E}_{Rest}$= restrain energy.

We used a recent ReaxFF article by Kowalik et al.^53^. That improved the earlier force fields for the more precise mechanical behavior of graphene, including stability of 5-, 6-, and 7-member rings. In addition, due to recent enhancements to the force field, studying LIG formation using ReaxFF is now a viable option. Readers are referred to the original ReaxFF papers for a more detailed description.

## Experimental section

### Synthesis of laser-induced graphene on cellulose and lignin

**Figure 3:**
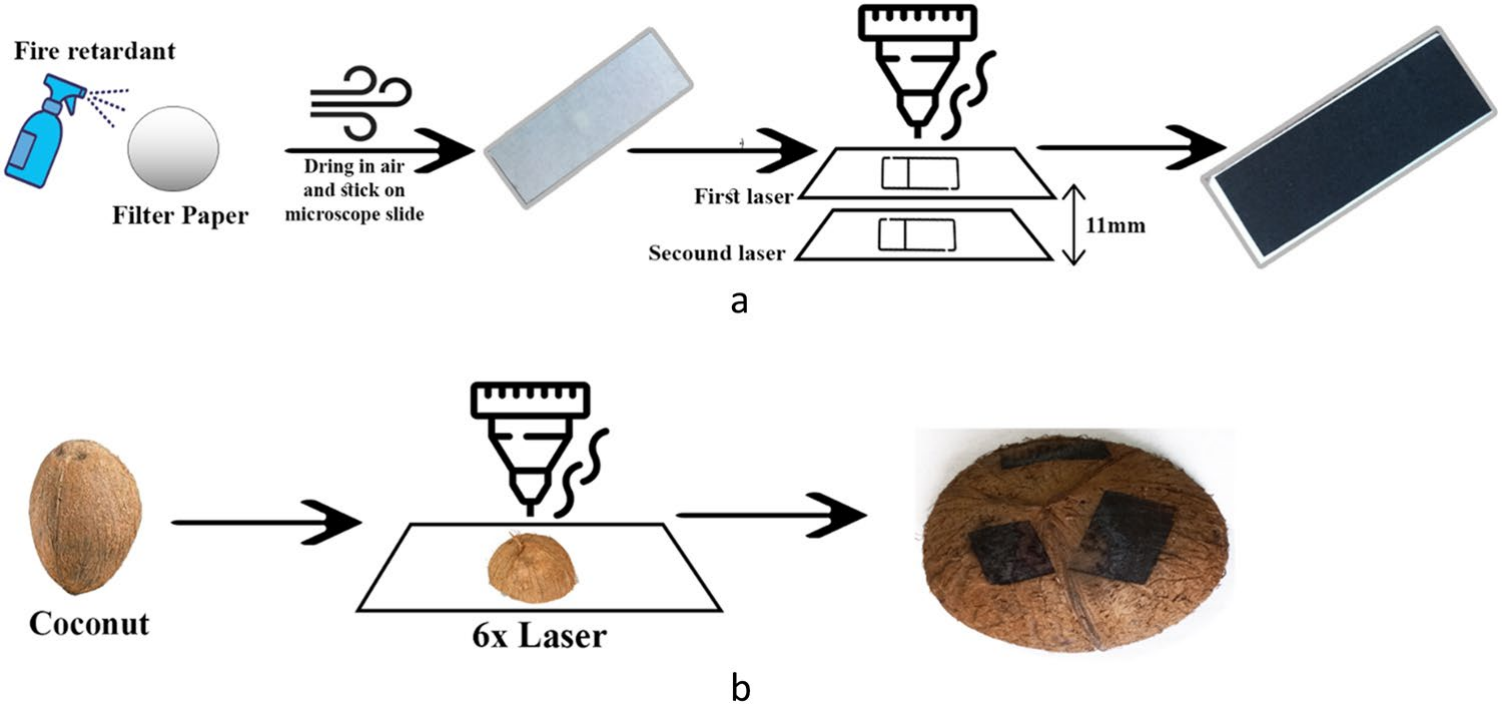
The schematic of the LIG process on (**a**) Filter Paper and (**b**) Coconut.

Whatman filter, was sprayed with a commercial phosphate-based fire retardant, and then exposed to ambient air flow for 24 h to dry after that, the paper substrates were flattened and tapped on a ~ 1.4 mm thick glass Microscope slide, and subsequently irradiated by an OMTech 60w CO_2_ laser engraver cutting machine (10.6 µm) continuous-wave. Large amounts of fire retardant did not significantly affect the results. The laser was set to line scan swing mode with a line spacing of 0.1 mm. The filter sheets were lasered with a speed of 80 mm/s and a power of 8%, first around 11 mm below the focal plane and then lased with the same parameters, at the focal plane. Figure 3a illustrates the cellulose LIG synthesis process.

Figure 3b shows Coconut LIG manufacturing steps, Coconut (Cocos nucifera) shells obtained from a local store were washed, dried, and broken into smaller pieces. The coconut shell was initially laser-treated with a power of 7% at a speed of 50 m/s, which caused it to transform into amorphous carbon. The additional lasering stages were then carried out with the same power and speed (6 times). To reduce focus changes with the coconut shell laser, it’s crucial to place a relatively flat region of coconut under the laser.

## Result and discussion

### General observations in MD simulations

As the primary indicator of graphene formation and graphitization throughout the reaction is the quantitative and qualitative analysis and analysis of the generated carbon rings, we have illustrated the number of 5-, 6-, and 7-member rings during the simulation. During the simulation, the formation of 3-, 4-, or 8-member rings, which were unstable, was also observed.

Since a significant amount of gas, including H_2_, CO, and H_2_O, is generated during laser operation, LIG has high porosity, as was previously indicated.

LIG sheets were separated from other molecules and cooled to 300 K. The radial distribution function (RDF) for carbon atoms was then calculated. The radial distribution function (RDF) is a key tool for analyzing the structure and properties of molecular systems in molecular dynamics (MD) simulations. The RDF is a probability distribution that describes the density of atoms or molecules at different radial distances from a central atom or molecule. The RDF provides important information about the spatial arrangement of particles in the system, as well as the types and strengths of intermolecular interactions.

**Figure 4:**
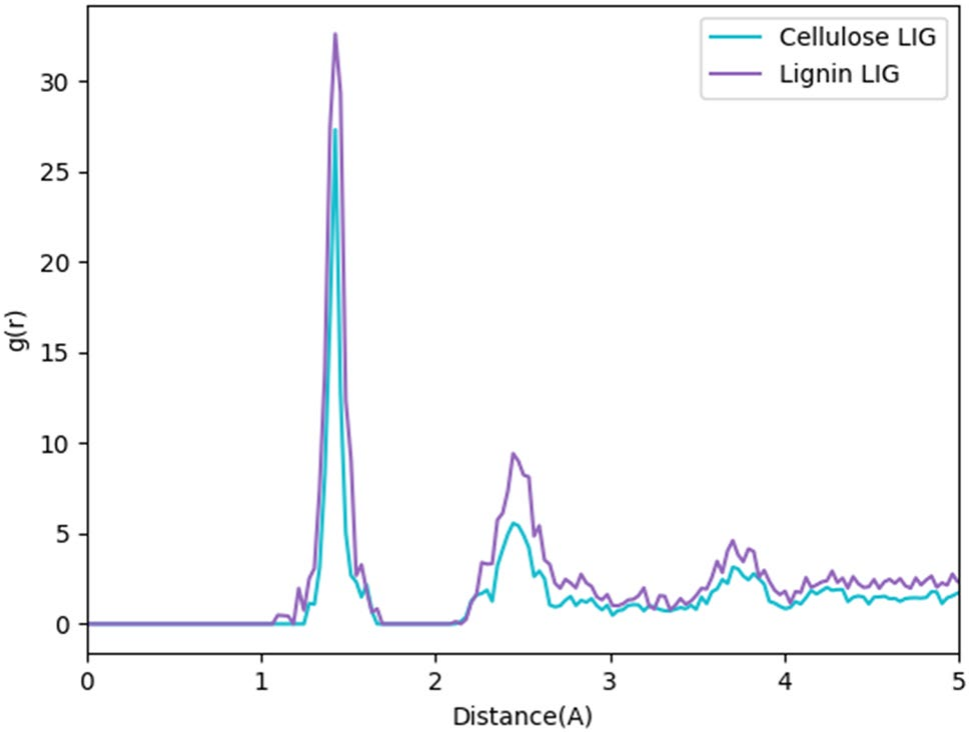
RDF for cellulose and lignin LIGs.

Figure 4 compares the radial distribution functions estimated between two carbon atoms for cellulose and lignin. For the RDF of LIG formed from cellulose and lignin, a peak is seen at 1.4. The peak’s wide base around 1.2 and 1.6 indicates that the examined structures contain bonds besides sp2–sp2 links. And the second peak (between 2.3 and 2.8) relates to the carbon ring's nearest neighbor. Figure S1 Illustrates the temperature variation in simulation Duration.

### Experimental results and characterization

To confirm that our lasing process produces LIG, we characterize our laser-etched samples with Raman spectroscopy. Raman spectroscopy provides information about the LIG composition in terms of the number of atomic layers and defect density.

The D peak (1330 cm^−1^), G peak (1600 cm^−1^), and 2D peak (2700 cm^−1^) are the three characteristic peaks of graphitic structures in Raman spectroscopy^54,55^. The ratio of D to G peak intensity can indicate the existence of defects, such as edges and functional groups, and the D band suggests bent graphene sheets or other defect sites.

**Figure 5:**
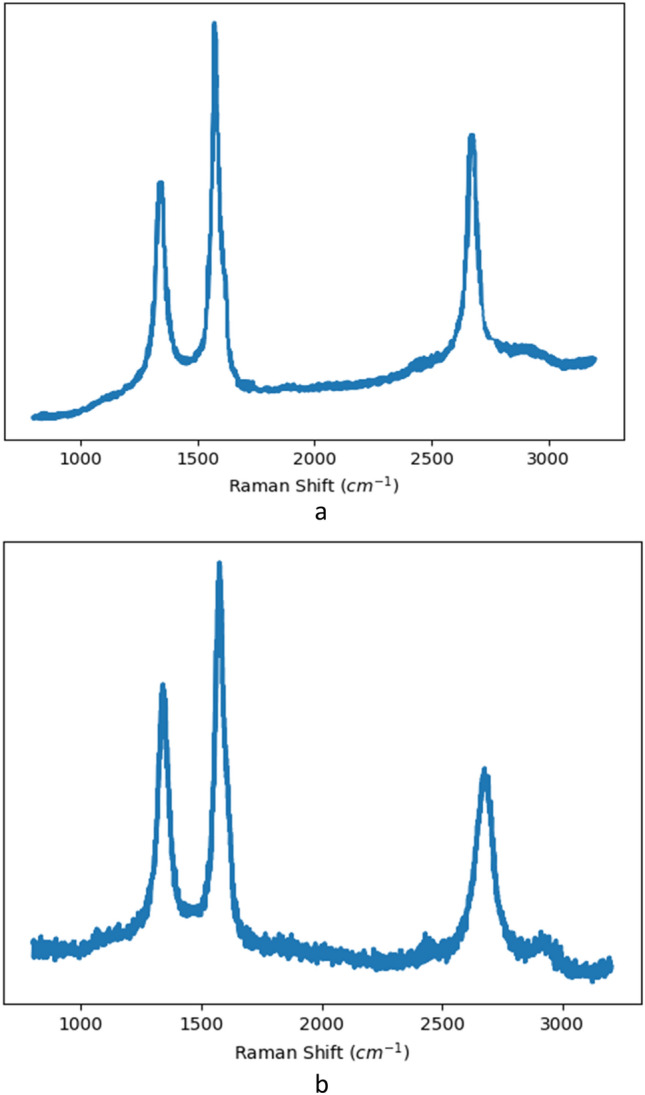
Raman spectrum for LIGs formed from (**a**) Coconut Shell and (**b**) Filter paper.

Since aliphatic carbon molecules are more reactive than aromatic carbon^19^, hemicellulose and cellulose are more rapidly decomposed and yield more defects during laser irradiation. Figures 5a and b show Raman spectrums of cellulose LIG and lignin LIG, The ID/IG ratio for paper LIG (~ 0.75) is larger than Coconut LIG (~ 0.61), indicating that the defect density is lower in coconut LIG compared to Paper LIG. As a result, a greater aromatic lignin concentration is more conducive to the formation of fewer graphene defects in the produced LIG.

**Figure 6:**
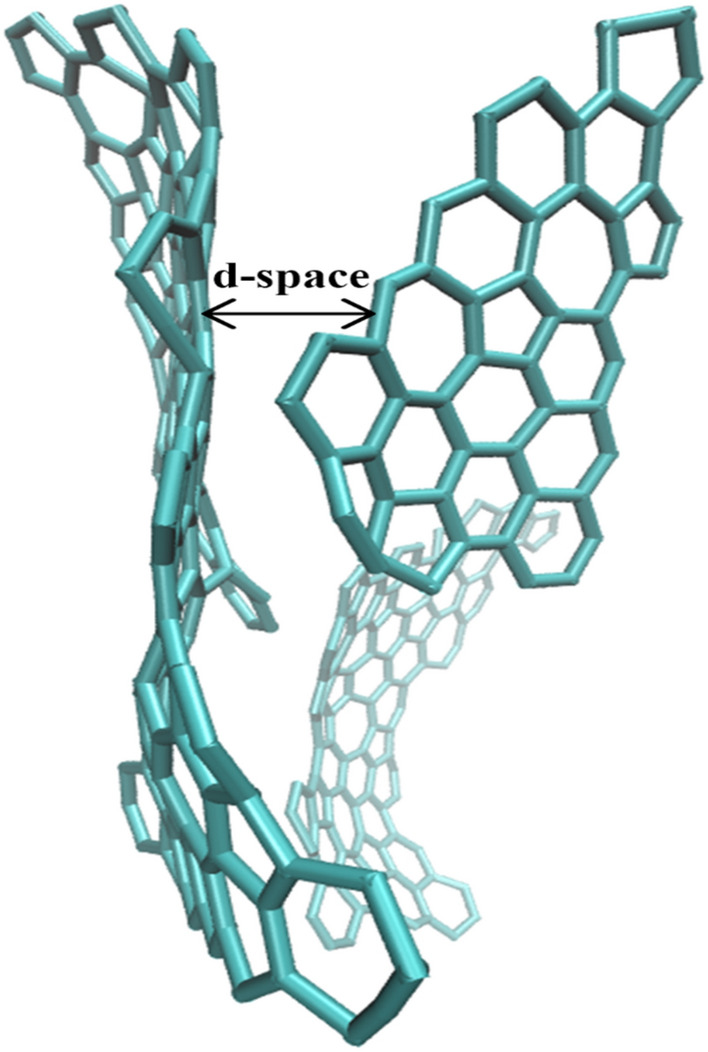
The Molecular structure illustrates the d-spacing(∼ 3.3–3.4 Å) among two planes for lignin LIG.

Figures 6 illustrates several five and six-membered rings in simulation time duration. The simulation results show that the ratio of pentagonal to hexagonal rings ($\frac{{r}_{5}}{{r}_{6}}$) as an indicator of the presence of defects in the graphene sheet is higher in cellulose ($\frac{{r}_{5}}{{r}_{6}}$=0.46) than in lignin ($\frac{{r}_{5}}{{r}_{6}}=0.42$), which is the same as the Raman results.

Higher-resolution TEM (HRTEM) images of coconut LIG demonstrate that the flake is composed of a few layers of graphene, revealing clear graphene fringes with the usual 0.34 nm d spacing^18^. Spacing between the subsequent graphitic layers was determined from the ReaxFF simulations performed in this study to be between 3.4 and 4.0 (Fig. 6), which is in line with the Experimental Results.

**Figure 7:**
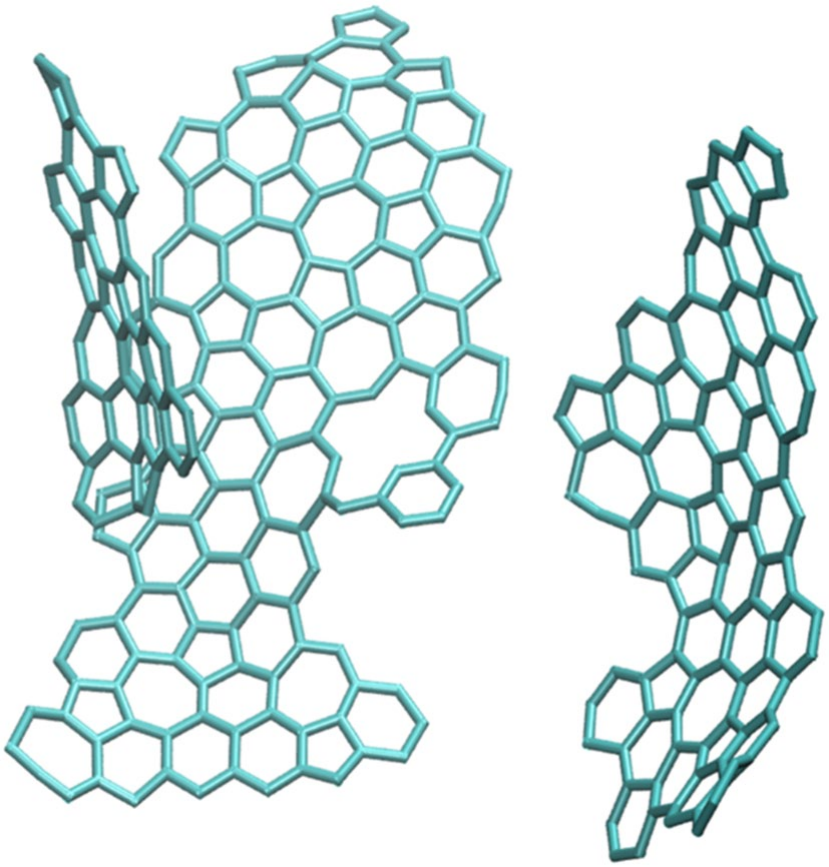
Lignin LIG molecular structure from MD that is completely in adoption with TEM image of LIG that shows a heptagon with two pentagons as well as a hexagon.

Figure 7 illustrates the structure of lignin-derived LIG from MD. This structure's existence of several defects and surface wrinkles is compatible with experimental results. In recently published works such as^11^, TEM image of an area of the LIG surface with pentagonal and heptagonal rings could be indicated.

The graphene surface has a large surface area due to its hierarchical structure and the presence of numerous wrinkles. LIG’s carbon has a hexagon and pentagon-heptagon hybrid lattice. Since there is no time for equilibration to the conventional hexagon lattice due to the quick cooling after laser irradiation.

For each simulated polymer, the LIG yield is determined by dividing the LIG molecular weight by the total molecular weight of all the simulation cell atoms. Table 1 shows the LIG yield for cellulose and lignin, with lignin producing more LIG than cellulose.

**Table 1:** Simulation cell details and determined LIG yield.

Initial structure name	Molecular formula	NO of monomers	Cell size (A)	Density($\frac{{\text{g}}}{{{\text{cm}}}^{3}})$	LIG yield
Cellulose	C_6_H_10_O_5_	60	24.7 × 24.7 × 24.7	1.07	0.23
Lignin	C_81_H_92_O_28_	7	32.7 × 32.7 × 32.7	0.49	0.34

To calculate the LIG yield for each polymer, we divide the molecular mass of LIG (${m}_{LIG}$) by the total molecular mass of all the atoms (${m}_{Total}$) used in the simulations. This calculation is performed using Eq. (2).

2 $$LIG Yield= \frac{{m}_{LIG}}{{m}_{Total}}$$

### Analysis of formed rings and surface area

In the laser-induced graphene (LIG) process, the formation of carbon rings within the graphene sheets is an important consideration for the electronic properties and mechanical stability of the resulting material. When using cellulose and lignin substrates, the laser ablation process results in the formation of sp2 hybridized carbon atoms that arrange themselves into hexagonal rings, the building blocks of graphene. These rings are crucial for the formation of graphene sheets, which are responsible for the high electrical conductivity and unique mechanical properties of LIG. The size and distribution of the carbon rings within the graphene sheets can affect the electrical conductivity and mechanical properties of LIG. The presence of defects, such as disordered rings, can affect the mechanical stability of LIG, while larger carbon rings can result in a decrease in electrical conductivity due to the presence of sp3 hybridized carbon atoms.

As can be observed, the primary structure of lignin consists of hexagonal rings (Fig. 2c). First, these rings begin to open and their number decreases quickly, converting lignin into amorphous carbon and after that, the formation of graphene sheets begins.

**Figure 8:**
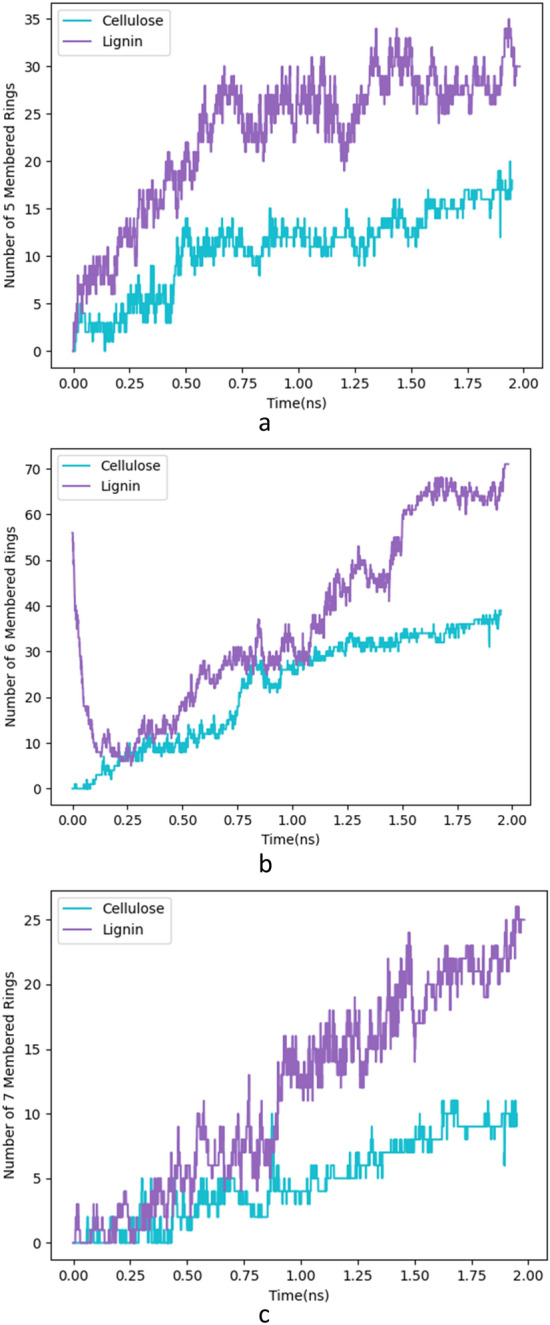
The number of different rings formed duration simulation time. (**a**) 5 membered Rings (**b**) 6 membered rings (**c**) 7 membered rings.

Also, Fig. 8b and c illustrate 5 and 7-member ring evaluation during simulation. The 5-member and 7-member rings are produced during the simulation process since they are absent at the beginning of the process and in the primary structures of cellulose and lignin. The presence of these rings and defects enhances the electrical conductivity of graphene.

Experimental TEM imaging may be used to identify these peculiar rings, which are present in LIG in contrast to pristine graphene. These pictures also confirm the existence of these rings in LIG, which are the consequence of the fast synthesis and sudden temperature variations.

As said, one of the applications of LIG is in catalysts and sensors that require a large surface area. We estimated the LIG's surface area at every time step.

**Figure 9:**
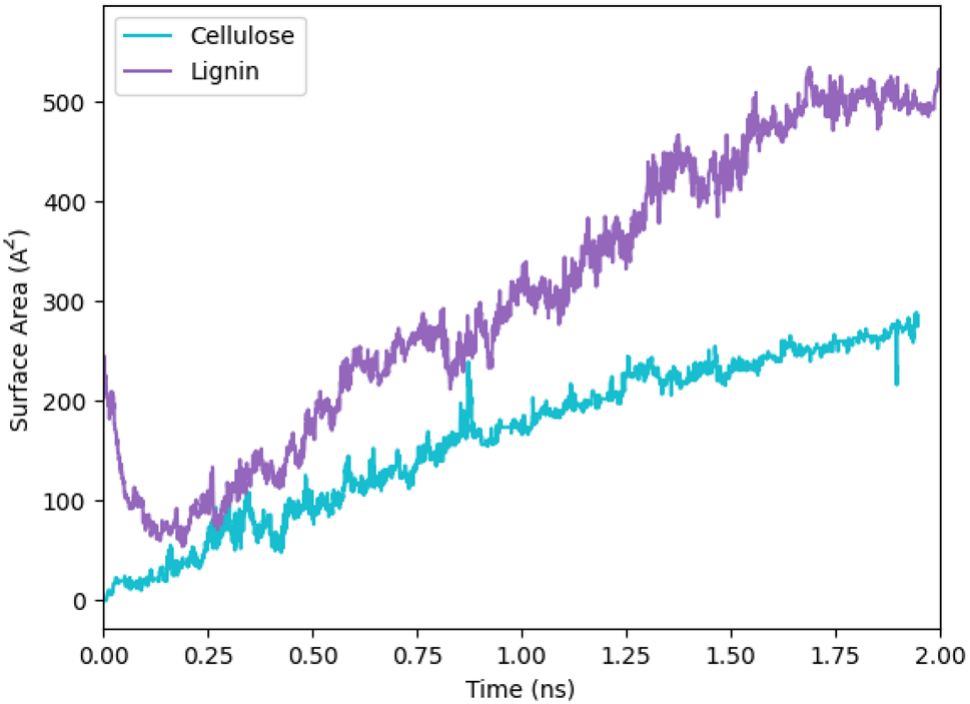
Calculated surface area of LIG from lignin and cellulose.

Figure 9 illustrates the evolution of the surface area of produced LIG in simulation Time. The surface area is calculated by adding the areas of all 3–8-member carbon rings that exist at each time step. In addition, the LIG surface area is directly related to the 5-, 6-, and 7-member rings. It is important to know all these measurements were done per simulation cell size.

### Simulated products distributions

**Figure 10:**
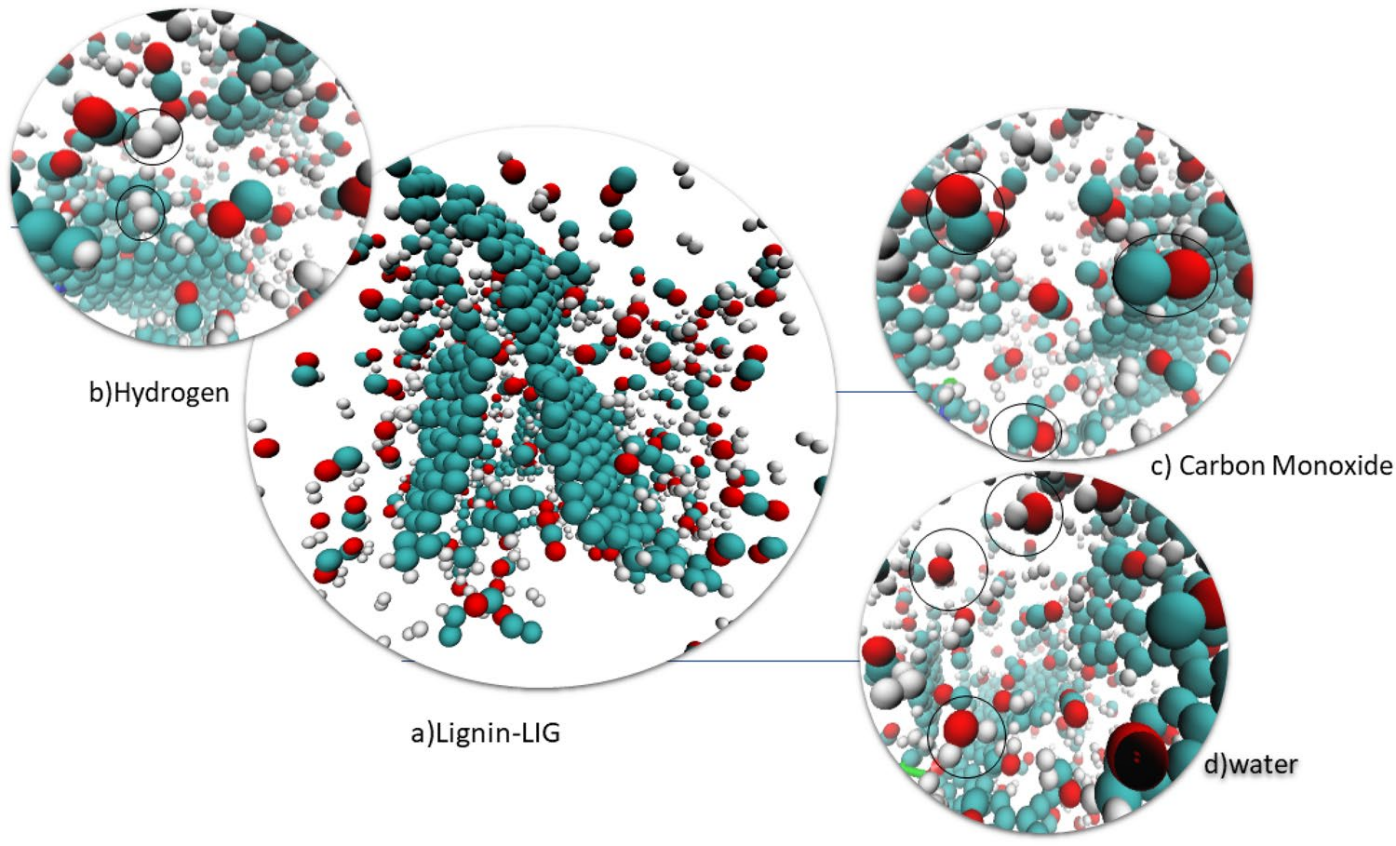
Visualization of simulation and gasses evolved during the process, cyan particle as carbon, white as hydrogen, and red as oxygen.

One key aspect of the LIG process is the generation of liberated gases, which are produced during the laser ablation of the substrate material. In the case of cellulose and lignin substrates, the liberated gases include carbon monoxide, carbon dioxide, and water vapor. These gases play a crucial role in the formation of LIG, as they help to create a porous structure within the substrate material, allowing for the formation of graphene sheets. Figure 10 illustrates the simulation and gasses that evolved during the process.

**Figure 11:**
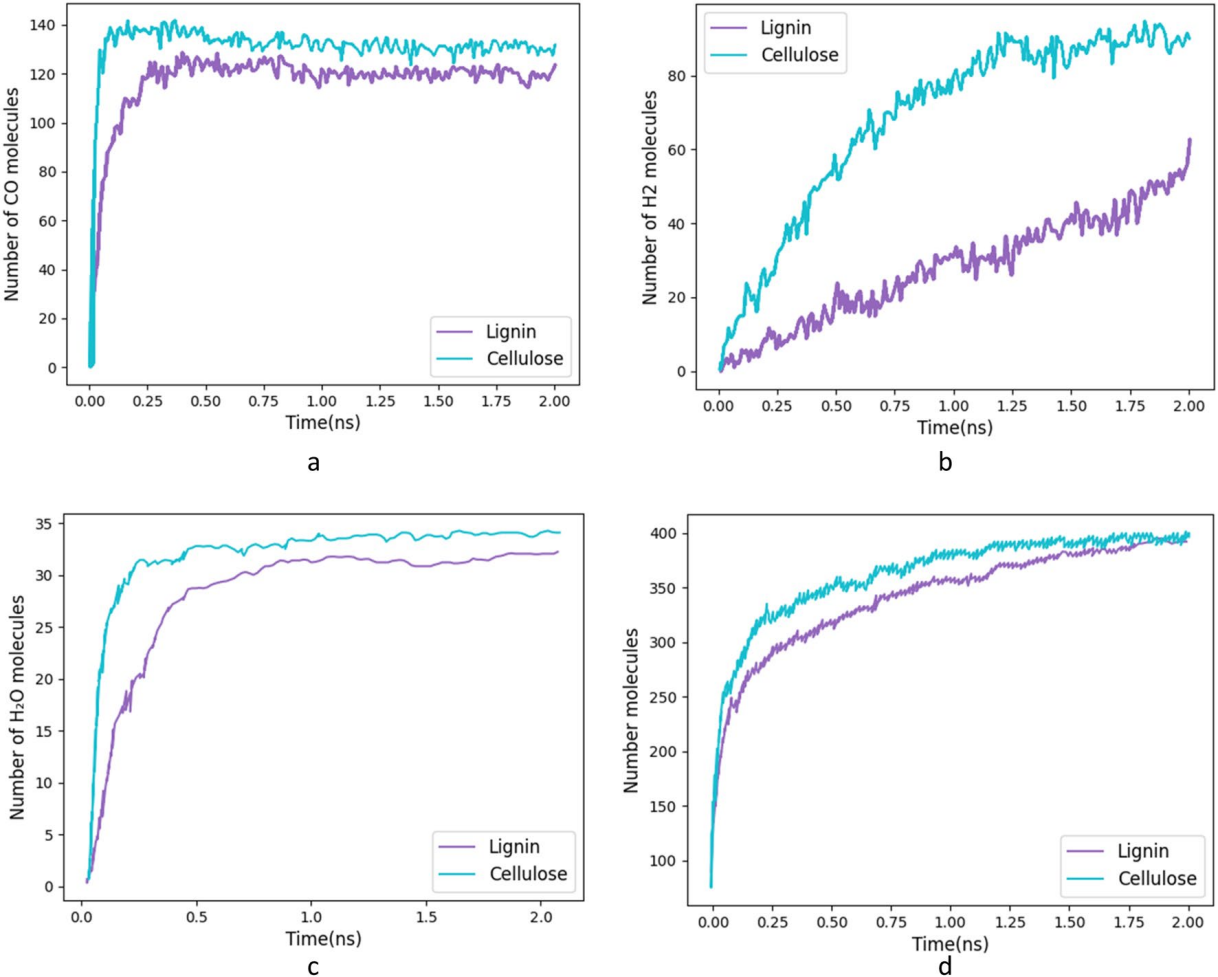
Produced Species in Simulation Time. (**a**) Number of CO molecules (**b**) Number of H_2_ molecules (**c**) Number of H_2_O molecules (**d**) Total molecules Number.

The liberated gases also play a role in the doping of the graphene sheets, influencing their electrical conductivity. The formation of these gases during the simulation process is illustrated in Fig. 11. During both processes, H_2_O, CO, and H_2_ were generated, while hydrocarbons such as C_2_H_2_, C_2_H_4_, and C_6_H_6_ and some C_x_H_y_ were also observed. The emission of gases during the evolution process is related to the porosity of LIG. Figure 11b shows the number of H_2_ molecules that are emitted in simulation time, which evolves steadily as the LIG transforms into an orderly structure.

Figure 11a shows the number of CO-produced molecules, Due to the larger mass percentage of oxygen in cellulose monomer (49%) compared to lignin monomer (29%), the amount of CO emitted for cellulose is more. that CO is produced quickly during the first conversion of cellulose and lignin into an amorphous media.

During the transition of amorphous carbon to graphene, the process of graphitization for both cellulose and lignin polymers leads to the release of water vapor that lasts for around 0.5 ns before becoming constant as seen in Fig. 11c. In the case of carbon dioxide, this production stop time is about 0.2 ns. Additionally, hexagonal rings start to form during this process. The carbon–carbon bond is broken over time, resulting in the release of carbon and oxygen and an increase in the amount of carbon monoxide. Since the hydrogen–oxygen bond has more energy, it takes longer for the number of water vapor molecules to become constant.

The presence of oxygen in the polymer monomers results in the bonding of carbon and oxygen, forming CO gas. The specific molecular changes in the polymer backbone influence the types of gases produced during the formation of LIG. By comprehending the molecular composition of the polymer, we can predict and control the evolution of gases during the LIG process, thereby tailoring the properties of graphene.

According to reports, raising the experiment lasing power would enhance the amount and rate at which gases were released. In this study and previous studies, it was demonstrated that decreasing the simulation temperature (below 3000 K) causes the formation of amorphous carbon (Fig. S3a), whereas increasing the simulation temperature (above 3000 K) results in the formation of separate species. (Fig. S3b).

## Conclusion

In this study, we discussed the use of laser irradiation to produce graphene structure with molecular dynamics simulation with LAMMPS and REAXFF for the LIG synthesis mechanism on two eco-friendly substrates, cellulose and lignin. Our simulated molecular structure agrees with recent experimental findings in the literature. cellulose and lignin, two abundant biopolymers, have attracted interest as potential substrates for LIG synthesis and the use of lignin and cellulose as substrates for LIG synthesis not only offers a sustainable and cost-effective route for graphene production but also presents an opportunity to valorize lignin and cellulose waste materials.

An amorphous structure is formed during the initial 0.2 ns of the simulations that grows into an ordered graphitic structure with 5-, 6-, and 7-membered rings. This study aims to understand the chemical and physical processes involved in LIG synthesis on cellulose and lignin substrates. Paper LIG and coconut LIG are two different kinds of characteristics. Compared to Coconut LIG, Paper LIG has a higher ID/IG ratio, which suggests fewer defects. Additionally, cellulose has higher pentagonal to hexagonal ring ratios (r_5/r_6) than lignin, which suggests fewer graphene defects in the LIG that are formed. The Raman results are the same as this.

In contrast to H_2_, which evolves steadily as the LIG transforms into an orderly structure, it has been noticed that CO is produced quickly during the first conversion of cellulose and lignin into an amorphous structure. Additionally, we found that the surface area for LIG generated from lignin is higher than cellulose LIG. These findings contribute to the current understanding of the LIG synthesis mechanism and can facilitate the development of efficient and sustainable methods for large-scale graphene production.

We aim to validate existing models for sustainable materials and develop molecular-level insights. It also sets the stage for future studies on LIG's molecular dynamics and exploration of its mechanical, thermal, and physical properties, The use of different ReaxFF potentials may provide valuable insights and enable comparisons among different types of LIG.

## Methods

### Materials

Whatman Grade 40 hardened ashless cellulose filter paper (95 g m^−2^, 210 μm of thickness, 0.007% ash content), commercial fire retard (Flametect C-WD, UK), normal coconut shell (were mature tall variety of brown fruits), microscope slide and some other usual laboratory devices were utilized.

### Synthesis of LIG on cellulose

Whatman filter, was sprayed with a commercial phosphate-based fire retardant, and then exposed to ambient air flow for 24 h to dry after that, the paper substrates were flattened and tapped on a ~ 1.4 mm thick glass microscope slide, and subsequently irradiated by an OMTech 60w CO_2_ laser engraver cutting machine (10.6 µm) continuous-wave. The laser was set to line scan swing mode with a line spacing of 0.1 mm. The filter sheets were lasered with a speed of 80 mm/s and a power of 8%, first around 11 mm below the focal plane and then lased with the same parameters, at the focal plane. The coconut shell was initially laser-treated with a power of 7% at a speed of 50 m/s, which caused it to transform into amorphous carbon. The additional lasering stages were then carried out with the same power and speed (6 times). To reduce focus changes with the coconut shell laser, it's crucial to place a relatively flat region of coconut under the laser.

### Supplementary Information

Supplementary Information 1.

Supplementary Video 1.

Supplementary Video 2.

## Data Availability

The datasets used and analyzed during the current study are available from the corresponding author upon reasonable request.
