# Supplementary material for: Simulation and experimental evaluation of laser-induced graphene on the cellulose and lignin substrates
Source: Sci Rep. 2024 Feb 23;14:4475. doi: 10.1038/s41598-024-54982-1 (PMC10891141; doi:10.1038/s41598-024-54982-1)
Supplement: Supplementary file 1 — Supplementary Information 1. [file 41598_2024_54982_MOESM1_ESM.docx]

**Supplementary Materials for:**

**Simulation and Experimental Evaluation of laser-induced graphene on the Cellulose and Lignin substrates**

**Ali Ghavipanjeh^1^, Sadegh Sadeghzadeh^2*^**

1- MSc of Nanotechnology Engineering, School of Advanced technologies, Iran University of science and technology, Tehran, Iran

2,*- Associate Professor of Nanotechnology Engineering, School of Advanced Technologies, Iran University of Science and Technology, Tehran, Iran, [sadeghzadeh@iust.ac.ir](mailto:sadeghzadeh@iust.ac.ir)

**Movie S1:** Molecular structure of LIG formed from Lignin at 3000 K Carbon atoms are shown as cyan.

**Movie S2:** Molecular structure of LIG formed from Cellulose at 3000 K Carbon atoms are shown as cyan.

| **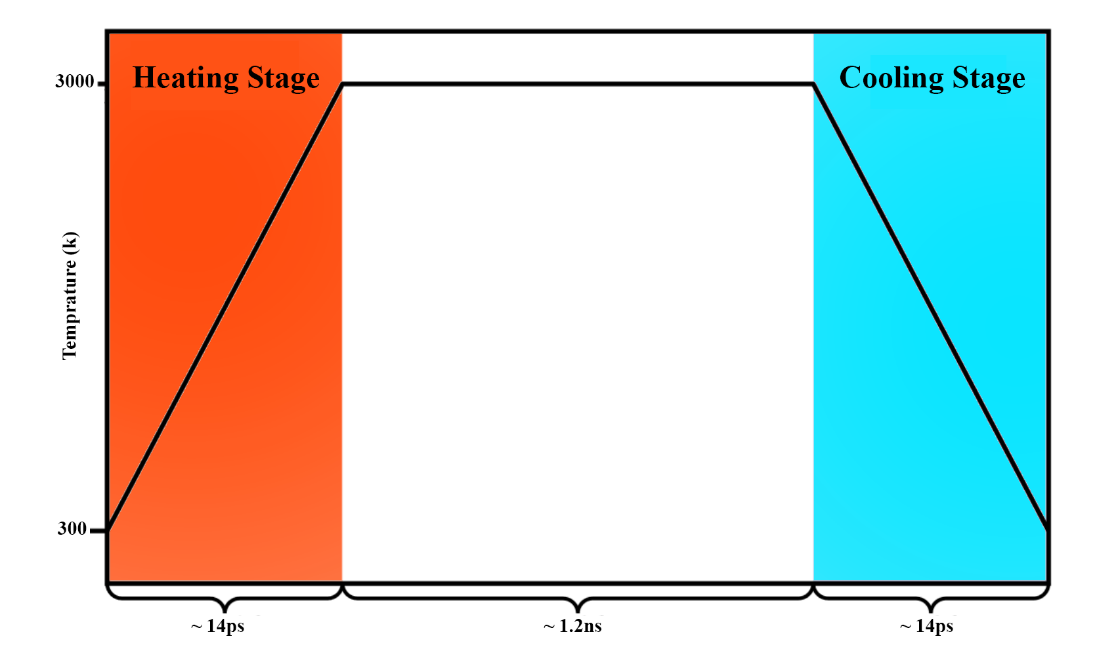** |
| --- |

**Fig. S1.** Temperature variation in simulation Duration Time

**Fig. S2.** Molecular Dynamic Simulation Stages

| **a)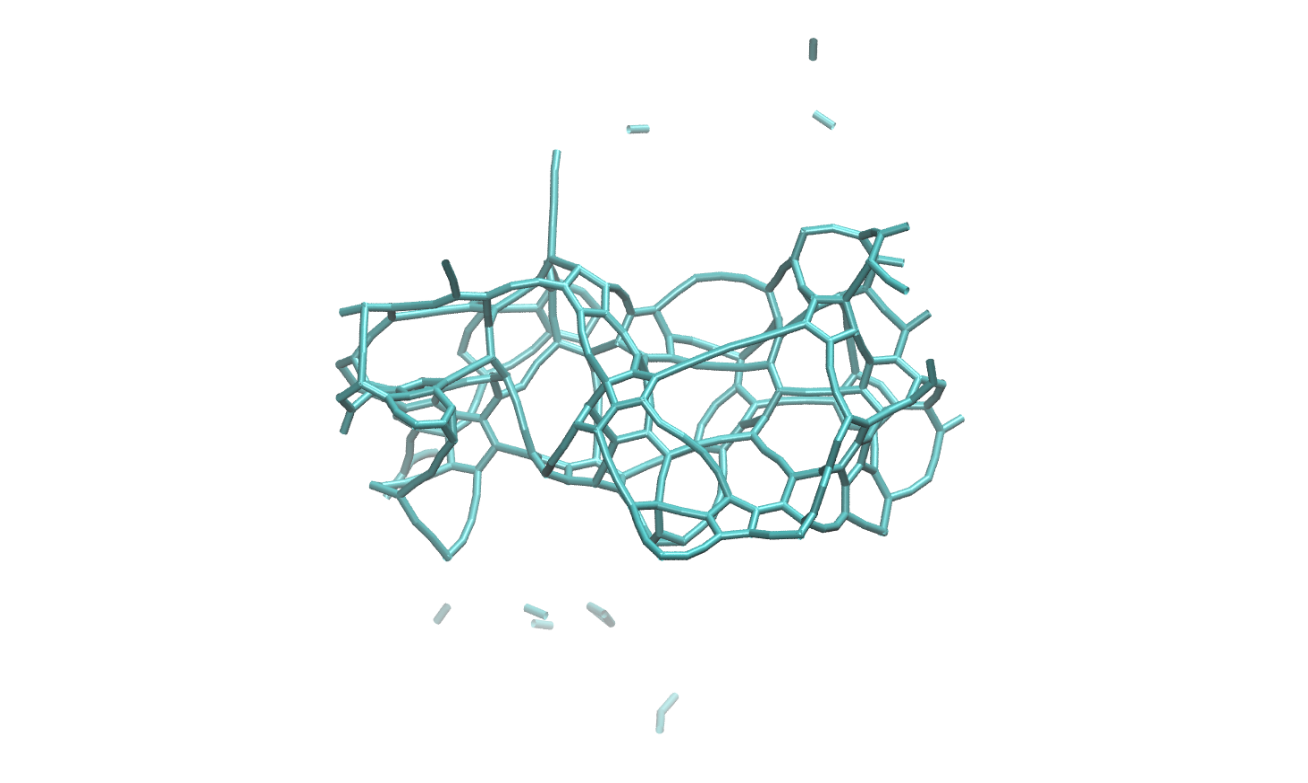** | **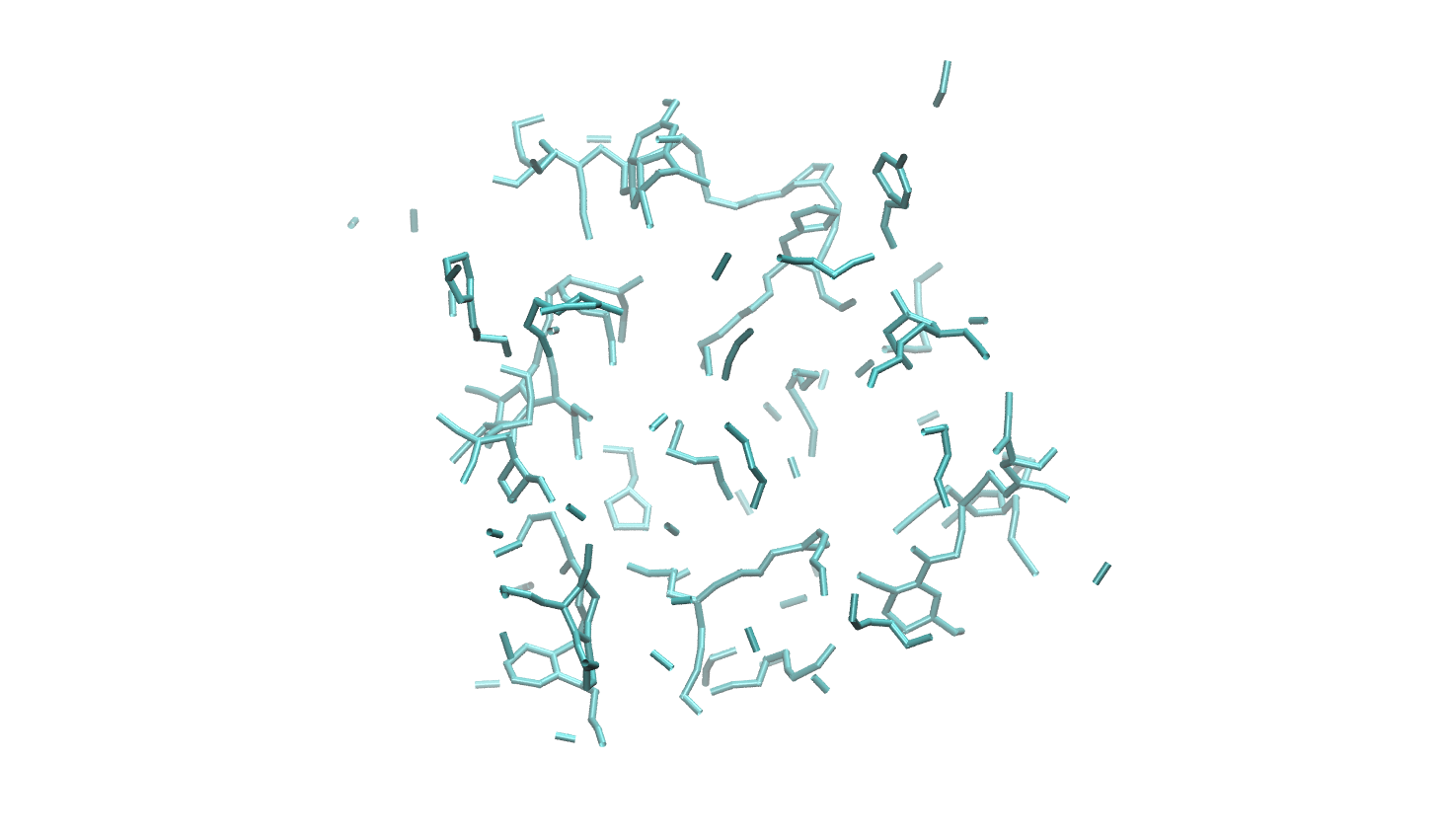b)** |
| --- | --- |

**Fig. S3.** Final Molecular structure of Lignin LIG in a)2500 K. b)4000K
